# “Reddit’s dark knight”: moral inversion, digital populism, and the aesthetic of resistance

**DOI:** 10.3389/fsoc.2026.1780129

**Published:** 2026-04-22

**Authors:** Anurag Shekhar, Musawenkosi D. Saurombe

**Affiliations:** Department of Industrial Psychology and People Management, College of Business and Economics, University of Johannesburg, Johannesburg, South Africa

**Keywords:** class conflict, digital populism, meme activism, moral disengagement, Reddit discourse, vigilante justice

## Abstract

**Introduction:**

In December 2024, Luigi Mangione was arrested in connection with the killing of UnitedHealthcare CEO Brian Thompson. Rather than condemning the accused, large numbers of Reddit users celebrated him as a folk hero. This study examines how that construction emerged and what it reveals about public attitudes towards institutional trust and retributive justice online.

**Methods:**

A total of 6,466 comments across 38 threads from 14 subreddits, including r/politics, r/antiwork, r/WorkReform, and r/Fauxmoi, were collected between December 2024 and April 2025. Data were analysed using reflexive thematic analysis.

**Results:**

Five themes emerged: folk hero glorification and moral inversion; distrust in institutions and folk justice; systemic injustice, moral outrage, and catharsis; digital protest and anti-corporate activism; and the spectacle and aesthetic of resistance.

**Discussion:**

These themes show how digital communities construct alternative moral orders that invert formal notions of justice and elevate grassroots counter-narratives. Drawing on digital populism, moral disengagement theory, and spectacle culture, the study explains how Reddit’s affordances, including anonymity, algorithmic amplification, and weak content moderation, intersect with healthcare injustice, class resentment, and institutional distrust. The findings are relevant for researchers, policymakers, and platform designers seeking to understand how retributive discourse circulates under conditions of eroded institutional trust.

## 1 Introduction

**Figure 1:**
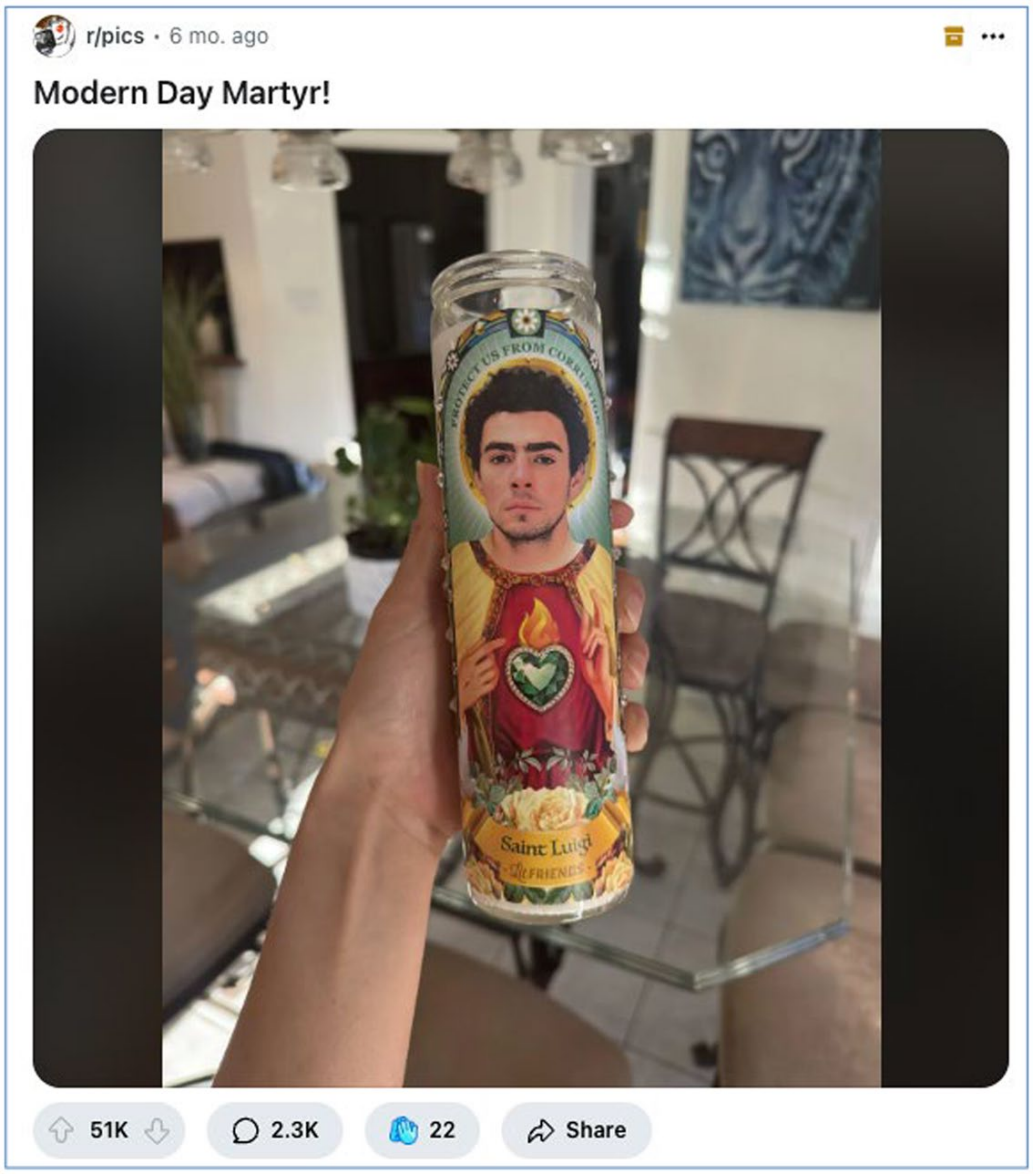
“Saint Luigi”: Memeified martyrdom and the aesthetic of digital resistance (June 2025, image retrieved from Reddit). A devotional-style candle image of Luigi Mangione, shared by a Reddit user on r/pics, represents the early visual memeification of Mangione as a modern-day saint. The image exemplifies how digital publics construct symbolic heroes through humour, reverence, and resistance. This figure sets the narrative tone for Mangione’s online transformation from alleged murderer to folk martyr.

In December 2024, the shocking assassination of UnitedHealthCare CEO Brian Thompson outside a Manhattan hotel in New York City, United States, sparked not only widespread media coverage but also an extraordinary and polarising reaction on social media (Basu, 2024; Notopoulos, 2025; Watercutter, 2024). The killing occurred against a backdrop of widespread public frustration with the American healthcare system, marked by chronic insurance denials, rising premiums, and a pervasive sense that corporate interests had come to outweigh human welfare (Economic Times, 2024; Levenson, 2025). It is within this socio-political context that a large segment of online users, particularly on Reddit, openly expressed support for the accused, Luigi Mangione, rather than the unanimous condemnation that might conventionally be expected (Schlott, 2025). Reddit threads rapidly filled with praise, sympathy, dark humour, and even admiration for Mangione (see Figure 1), portraying him as a symbol of resistance against a dehumanising healthcare system. This groundswell of sentiment challenges conventional expectations of moral condemnation for violent acts and highlights the growing role of digital platforms in shaping counter-narratives around justice, power, and corporate accountability (Basu, 2024; Suciu, 2024). This response invites analysis through the lens of the counter-public sphere—spaces where groups excluded from or disillusioned with dominant public discourse construct oppositional narratives and alternative moral frameworks (Fraser, 1990). As the literature review discusses, Reddit’s structural features make it a productive site for counter-hegemonic meaning-making—particularly when mainstream media and institutional narratives are seen as dismissing or suppressing popular grievance.

Reddit is conceptualised as a social media network and social news aggregator that describes itself as the “front page of the internet” (Brown et al., 2018; Robards, 2018). Structurally, the platform is divided into thousands of topic-based communities known as subreddits, which scholars conceptualise as a modular “community of communities” or a collection of digitally mediated “neo-tribes” (Massanari, 2015; Proferes et al., 2021; Robards, 2018). A core feature of this architecture is the voting system, which allows registered users to upvote or downvote content and comments to determine their level of prominence and visibility, with these interactions accumulating into “karma” points that de-emphasise individual identity in favour of the discourse itself (Proferes et al., 2021). Reddit’s governance structure is hierarchical, consisting of paid site administrators, volunteer moderators, and general users, with each subreddit governed by its own unique set of rules and norms rather than a single platform-wide content policy (Massanari, 2015; Proferes et al., 2021; Robards, 2018). This decentralised structure means that permitted speech varies considerably across subreddits. Local community norms shape what is acceptable, which has led scholars to critique the platform for hosting toxic technocultures where hostile political discourse can coexist alongside progressive and feminist communities (Massanari, 2015; Proferes et al., 2021).

While mainstream platforms such as YouTube, Facebook, TikTok and Instagram have visibly censored or restricted such discourse, often citing platform policies and reputational risks (Notopoulos, 2025), Reddit has emerged as a relatively less restrictive arena for users to voice raw, unfiltered reactions. Unlike identity-linked networks like Facebook or LinkedIn, Reddit affords users a high degree of perceived anonymity and pseudonymity, enabling what Suler (2004) terms the “online disinhibition effect”—a tendency for users to express more candid, intimate, or socially unacceptable views than they would in identity-linked or face-to-face settings (De Choudhury and De, 2014; Kahlow, 2024). Reddit’s architecture supports “dissociative anonymity” through throwaway accounts and pseudonymous profiles that allow users to separate their online actions from their offline identities, reducing the risk of professional or social retaliation (De Choudhury and De, 2014; Proferes et al., 2021). It is important to note, however, that this anonymity is perceived rather than absolute—Reddit is not free from data extraction or platform surveillance, but its structural affordances create conditions in which users behave as though they are (Brown et al., 2018; Triggs et al., 2021). In this sense, Reddit serves as fertile ground for studying the psychological, ideological, and emotional undercurrents that emerge when corporate leadership and systemic injustice intersect. Massanari (2015) situates Reddit within a broader participatory culture framework, noting that its affordances do not merely reflect public opinion but actively shape it—amplifying subcultural norms, enabling collective identity formation, and providing infrastructure for counter-hegemonic discourse that bypasses traditional media gatekeepers. In the context of the Mangione case, Reddit functioned specifically as a counter-public sphere in opposition to dominant institutional and mainstream media narratives (narratives that framed the killing as straightforward criminal violence rather than as a generalised or community-reinforcing space). This oppositional character distinguishes the discourse examined here from the broader variation in Reddit’s subcultural ecology, where community norms may reinforce rather than challenge dominant ideological formations.

The widespread support for Mangione, often couched in narratives of moral justice and popular resistance, reflects a deeper cultural pattern. Historically, figures who defied authority, especially when seen as striking back at unjust systems, have often been reimagined as heroes, even when their actions involved violence or criminality (Heering, 2021; Seal, 2009). Characters such as Bonnie and Clyde or Jesse James—figures deeply embedded in the American cultural imagination—were romanticised not for their crimes alone, but for their perceived defiance of banks, landowners, and state authority during periods of acute economic hardship (Milner, 2003; Stiles, 2012). These archetypes speak to a specifically American tradition of outlaw heroism rooted in class resentment and institutional distrust, a tradition that the Mangione case reanimates in a distinctly digital register. In the digital age, this archetype of the “outlaw-hero” has been reanimated and amplified through social media (Suciu, 2024). Platforms like Reddit enable such figures to be recast as symbols of rebellion, especially when they are seen as confronting exploitative systems (Schlott, 2025). In Mangione’s case, many users interpreted his actions not as cold-blooded murder, but as a desperate form of protest against a healthcare industry widely viewed as valuing profit over human life (Economic Times, 2024; Levenson, 2025). This reframing speaks to a collective yearning for justice in the face of institutional failure, one now intensified by the amplifying logics of platform algorithms, digital anonymity, and the viral power of online communities.

### 1.1 Purpose of the study

The purpose of this study was to explore how anonymous users on Reddit collectively framed and reframed the alleged killing of UnitedHealthCare CEO Brian Thompson by the accused, Luigi Mangione, ultimately transforming a criminal act into a narrative of folk heroism. Using a thematic analysis approach (Braun and Clarke, 2021), the study adopted a social constructivist lens to examine how digital discourse can generate collective meaning, emotional catharsis, and symbolic resistance in response to perceived systemic injustice.

This analysis focused on how Redditors, operating under conditions of perceived anonymity afforded by Reddit’s pseudonymous architecture, articulated outrage, vengeance, and empathy in highly affective and morally complex ways. In particular, we sought to understand how Mangione came to embody an emerging archetype of the *digitally-optimised outlaw*, a figure whose morally ambiguous actions are recast as righteous rebellion against corrupt systems.

Our theoretical framework integrates Digital Populism (Mudde, 2004), Moral Disengagement Theory (Bandura, 1999), and Spectacle Culture (Debord, 1967), and Content Moderation as a paradoxical platform response to explain how Reddit’s affordances, anonymity, algorithmic amplification, and weak content moderation intersect with broader structural antecedents such as healthcare injustice, class resentment, and institutional distrust. Within this context, platform dynamics do not simply reflect public opinion; they actively shape it through the creation, dissemination, and reinforcement of emotionally resonant, simplified, and aestheticised narratives.

## 2 A brief literature review

The convergence of social media, populist sentiment, and transgressive acts of extra-legal violence has fundamentally reshaped how digital publics engage with criminality and institutional authority (Gerbaudo, 2018; Ward, 2020). This pattern is well documented across multiple cultural contexts: in Norway and Canada, groups such as Soldiers of Odin Norge and La Meute leveraged social media to frame themselves as “watchful citizens” protecting communal values against perceived external threats (Castle and Parsons, 2019; Tanner and Campana, 2020); in Mexico, digital media assemblages positioned cartel leader El Chapo as a folk hero in direct opposition to government authority (Albarran-Torres and Goggin, 2023); and in the United States and India, vigilante groups broadcast spectacles of violence to reinforce nativist political discourses (Ward, 2020). Across these cases, digital platforms facilitate what Gerbaudo (2018) terms a “choreography of assembly,” enabling dispersed individuals to form online crowds dedicated to challenging liberal establishments or state failures. This digital response exemplifies what scholars term *digital populism*, a narrative framework that opposes the “virtuous people” to “corrupt elites” (Mudde, 2004), while drawing heavily on the mechanisms of *moral disengagement* (Bandura, 1999) and the concept of *spectacle culture* (Debord, 1967). The literature reviewed below provides the conceptual scaffolding for analysing how online communities may construct, aestheticise, and morally justify violence through folk hero narratives in a post-truth era.

It is worth distinguishing between discourse that simply reinforces existing community norms and discourse that actively challenges institutional narratives. The Reddit responses examined here reflect the latter. Users did not merely affirm shared subcultural values—they constructed an oppositional moral framework that challenged dominant narratives about crime, justice, and corporate power. Whether this reflects genuine ideological conviction or memetic participation is not straightforward. Klein (2015) notes that transgressive digital figures are typically cast as either vigilante heroes or malicious pranksters, while Albarran-Torres and Goggin (2023) observe that meme-based communication can generate a sense of solidarity even when the underlying positions are contradictory or fleeting. This study treats both responses—the earnest and the ironic—as meaningful rather than mutually exclusive. This is why Reddit is positioned here not as an echo chamber, but as a counter-public sphere where institutional authority is openly contested.

### 2.1 Digital populism and anti-elite narratives online

Populism is widely understood as a rhetorical framework that divides society into two antagonistic groups: a morally pure and victimised “people” versus a corrupt and self-serving “elite” (Mudde, 2004). Online platforms allow anti-elite narratives to spread widely, mobilising emotions and symbols that portray institutional leaders as malevolent and the common public as morally righteous (Prior, 2021). Research on digital populism observes that social networks amplify populist rhetoric, often merging with disinformation in *post-truth* environments (Gil De Zúñiga et al., 2020; Gründl, 2022; Prior, 2021; Sánchez-Holgado, 2025). While social media platforms broadly facilitate populist discourse, Reddit’s specific platform architecture makes it a particularly fertile environment for anti-elite narratives (Brown et al., 2018; Massanari, 2015). Its organisation into thousands of topic-based subreddits—conceptualised by Robards (2018) as digitally mediated “neo-tribes”—allows for the cultivation of insular community norms away from mainstream public scrutiny. Its voting system creates powerful echo chamber dynamics that prioritise the discursive interests of specific demographics, while its decentralised governance structure, managed by volunteer moderators with significant autonomy, has been shown to facilitate the rise of “toxic technocultures” alongside feminist counter-publics (Massanari, 2015; Proferes et al., 2021). Unlike identity-linked platforms where real-name policies constrain self-disclosure, Reddit’s affordance of anonymity and pseudonymity produces a disinhibition effect that enables uninhibited anti-establishment expression (Brown et al., 2018; Kahlow, 2024). These platform-specific dynamics (rather than populist sentiment alone) explain why Reddit in particular, rather than social media generally, became the primary arena for the discourse examined in this study (Diehl and Bargetz, 2023; Gerbaudo, 2018; Gründl, 2022).

This online populism often operates within what has been termed a *post-truth* environment, where appeals to emotion and personal belief outweigh verifiable facts in shaping public opinion (Prior, 2021). The term “post-truth,” named Oxford’s Word of the Year in 2016, refers to situations where emotional appeals and personal beliefs take precedence over objective facts in shaping public opinion. It involves the deliberate manipulation of reality to evoke emotions and shape people’s thoughts and feelings about social issues (Gil De Zúñiga et al., 2020; Prior, 2021).

### 2.2 Moral disengagement and justifications of vigilante violence

Bandura’s (1999) *moral disengagement theory* offers a powerful framework for understanding how individuals reconcile their support for violence with their own moral standards, particularly in online settings where anonymity reduces social accountability. This theory outlines mechanisms such as moral justification, euphemistic labelling, advantageous comparison, displacement of responsibility, distortion of consequences, and dehumanisation, cognitive tools that allow individuals to rationalise harmful acts as acceptable or even praiseworthy (Bandura, 2011). Critically, however, moral disengagement in digital contexts operates not merely at the individual level but as a collective, platform-mediated phenomenon. Trottier (2017) theorises digital vigilantism as a “weaponisation of visibility,” in which platform affordances (anonymity, algorithmic amplification, and mass coordination) enable communities to enact collective moral disengagement at scale, effectively separating target groups from the moral obligations usually owed to fellow citizens. This process frequently involves dehumanisation: in the context of nationalist digital vigilantism in China, Huang (2023) documents how intellectual women are branded as “traitors” or subjected to intense online harassment through discursive strategies that strip targets of their humanity to justify retribution. Similarly, Tanner and Campana (2020) show how algorithmic filter bubbles cultivate a sense of “moral sanctity” within far-right online communities, enabling members to frame acts of retribution as civic duty. What these cases share is the operationalisation of Bandura’s mechanisms not through individual cognition alone but through the collective architecture of digital platforms (i.e., upvoting, thread reinforcement, and community consensus) that normalise and reward moral disengagement as a social practise.

Across these contexts, online anonymity and emotional contagion foster digital echo chambers in which moral disengagement is not only tolerated but actively celebrated (Andoh, 2024). As Trottier (2017) notes in his analysis of digital vigilantism, online spaces increasingly facilitate collective rationalisations of punitive behaviour, where legal boundaries are dismissed in favour of “moral truths” shaped by group consensus and populist outrage. Social platforms like Reddit amplify such sentiment, reducing inhibitions and allowing users to validate each other’s justifications for extreme actions, what Bandura (1999) might call a collective disengagement environment.

### 2.3 Spectacle culture and the aestheticisation of justice

Another relevant lens is Debord’s concept of the *society of the spectacle*, which illuminates how sensational events are transformed into mediated spectacles and consumer entertainment (Debord, 1967). In Debord’s view, modern society converts lived reality into mere representations, where “all of life presents itself as an immense accumulation of spectacles,” displacing direct experience with images and narratives. Contemporary scholars have applied this idea to digital culture, noting that social media platforms turn news and conflicts into viral spectacles for mass consumption. Vigilante acts, in particular, become “attention-arresting visual acts of political discourse” when circulated online (Ward, 2020). Ward (2020) documents how images and videos of vigilante violence (such as live-streamed confrontations or graphic aftermaths) are disseminated to dramatise a cause, effectively turning private violence into a public performance. This spectacularisation serves both emotional and political purposes: it shocks and entertains audiences while also conveying a message or ideology in a visceral way. Such aestheticisation is a form of meaning-making: complex social realities (e.g., America’s healthcare failures) get condensed into symbolic, emotionally charged content that is easy to consume and propagate. Debord’s theory helps us recognise that the medium (social media spectacle) becomes part of the message; in high-profile criminal cases circulated online, the performance of outrage and heroisation can matter as much as the legal facts themselves.

The relationship between spectacle culture, digital populism, and moral disengagement is not incidental but structurally interconnected. Populism provides the ideological frame (people versus elite) that identifies who deserves to be celebrated or condemned. Moral disengagement provides the cognitive machinery that permits users to celebrate transgressive acts without experiencing moral conflict. And spectacle culture provides the aesthetic and affective register through which these reframings are performed, shared, and amplified. Together, these three mechanisms operate within and are accelerated by Reddit’s platform affordances—anonymity reducing inhibition, upvoting amplifying resonant content, and meme circulation extending reach across subreddit boundaries and into broader digital culture (Massanari, 2015).

Indeed, prior research demonstrates that true crime narratives consistently evolve into what commentators have termed “Ryan Murphy effect,” where a true crime story is dramatised and stylised until the vigilante appears attractive and relatable (Wiebe, 2025). This aligns with broader trends in spectacle culture: audiences are drawn to vigilante folk hero stories as a form of cathartic entertainment, blurring the line between righteous indignation and voyeuristic thrill. Prior research on digital media spectacles notes that online communities often participate in the co-creation of these narratives, adding iterative layers of humour, art, and myth (e.g., inside jokes, fan art, video remixes) that collectively mythologise real events (Albarran-Torres and Goggin, 2023). In the case of El Chapo, memes circulating on WhatsApp and Twitter during the period of December 2015 to July 2016 functioned simultaneously as artefacts of political contestation and as expressions of everyday survival humour, mixing folk legend with anti-government sentiment in ways that were geographically and temporally specific (Albarran-Torres and Goggin, 2023). Similarly, the use of Viking archetypes by Soldiers of Odin Norge on Facebook between 2015 and 2017 allowed the group to mask extremist agendas within familiar cultural scripts, attracting members through aesthetic resonance rather than explicit ideological recruitment (Castle and Parsons, 2019). These cases establish that meme-based folk heroisation is a recurrent feature of digital vigilante discourse, operating across different national contexts, platform environments, and timeframes.

Through the lens of spectacle culture, we understand that the social media environment encourages users not just to discuss events but to perform their stances in visually engaging, emotionally resonant ways. In such cases, the line between political outrage and participatory pop culture becomes blurred. As Albarran-Torres and Goggin (2023) argue, social media spectacle culture creates shared meaning through collective remixing, where jokes, memes, and moral judgements coexist in a single thread. Taken together, these theoretical perspectives suggest that digital publics aestheticise justice—rendering it visually seductive, emotionally cathartic, and virally transmissible—in ways that warrant systematic empirical examination.

### 2.4 Content moderation as paradoxical platform response

Content moderation on Reddit is managed through a decentralised governance structure in which paid site administrators establish broad platform policies while volunteer moderators exercise significant discretion over community-specific norms and rituals (Massanari, 2015; Proferes et al., 2021; Robards, 2018). These local governance practises allow subreddits to function as digitally mediated “neo-tribes,” where the ultimate authority for interpreting and enforcing behavioural conventions lies with individual community leaders (Proferes et al., 2021; Robards, 2018). However, the suppression of transgressive speech can paradoxically amplify anti-establishment sentiment, as platform-led interventions and bans are often resisted by users who justify hostility through an ideological foundation of “freedom of expression” (Brown et al., 2018; Massanari, 2015).

This dynamic is further complicated by the “weaponisation of visibility,” wherein users coordinate the public denunciation of targets to enact a parallel form of justice that bypasses formal legal and platform safeguards (Trottier, 2017). Although administrators may eventually remove such content, the social consequences are frequently enduring and persist across the global digital landscape (Trottier, 2017). Furthermore, “toxic technocultures” often persist despite moderation structures because Reddit’s algorithmic architecture and voting systems tend to aggregate content in ways that prioritise the interests of dominant, often hostile, demographics (Brown et al., 2018; Massanari, 2015). Some communities evade suppression entirely by cloaking transgressive discourse in irony or humour, building a sense of belonging around shared irreverence while remaining relatively shielded from institutional scrutiny (Massanari, 2019; Proferes et al., 2021).

Within the theoretical framework proposed in this study, content moderation therefore functions not as a neutral corrective but as a constitutive element of folk hero narratives—platform suppression is reinterpreted by users as confirmation that powerful institutions are silencing dissent, thereby deepening anti-elite sentiment and amplifying the symbolic status of the figure being moderated.

### 2.5 Theoretical model

**Figure 2:**
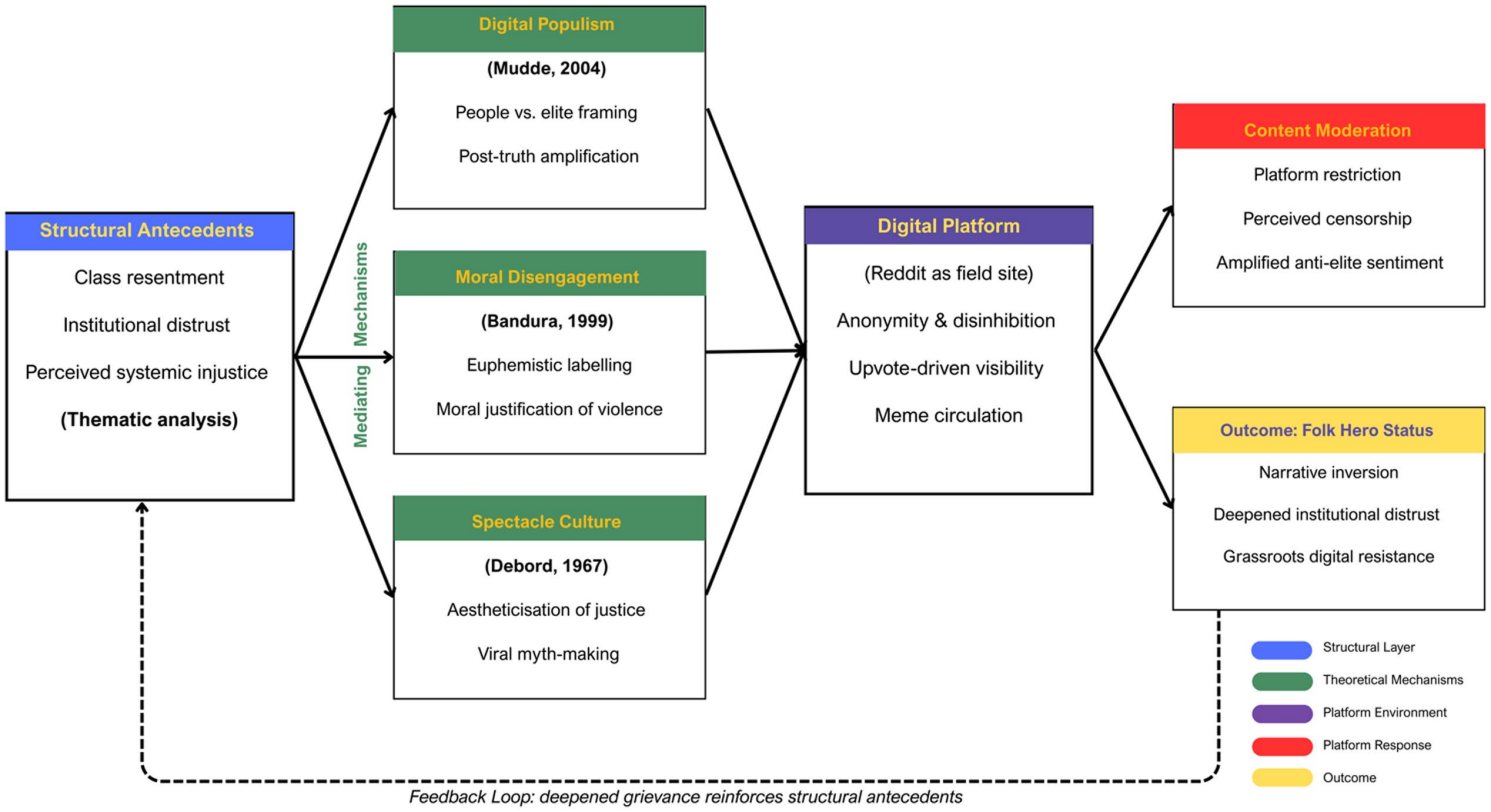
Theoretical framework (from structural grievance to digital folk hero status).

To fully understand the emergence of Luigi Mangione as a digital folk hero, this study proposes a multi-layered theoretical framework (see Figure 2) that integrates five key concepts: structural antecedents, digital populism, moral disengagement, spectacle culture, and content moderation, all operating within and amplified by the affordances of digital platforms like Reddit. Together, these mechanisms help explain how violent or criminal acts are re-narrated into emotionally and morally resonant stories of resistance, justice, and popular heroism.

At the foundation are structural antecedents, widespread socioeconomic grievances, institutional distrust, and perceived systemic injustice, which are consistently identified in thematic analyses of Reddit discourse. These grievances shape how users interpret acts of violence, particularly when the perceived victim represents a powerful or corporate elite. In this case, the healthcare system is viewed as exploitative and dehumanising.

Building upon this discontent, digital populism (Hameleers, 2020; Mudde, 2004) provides the discursive structure through which online communities frame the conflict as a battle between a morally pure, suffering “people” and a corrupt, insulated “elite.” Within this framework, accused individuals may be positioned as ordinary people striking back against entrenched power, their alleged acts reinterpreted as insurgent gestures within a broader moral struggle.

The concept of moral disengagement (Bandura, 1999) explains the cognitive processes that enable users to justify or celebrate vigilante violence. Mechanisms such as euphemistic labelling, advantageous comparison, and dehumanisation of victims allow communities to reframe harmful acts as morally necessary corrections to structural injustice.

Spectacle culture (Debord, 1967) further situates the case within the contemporary digital media environment, where justice becomes aestheticised and virality confers symbolic power. Memes, visual remixes, and dramatised narratives transform accused figures into iconic images of resistance, converting legal proceedings into participatory spectacle.

Content moderation operates as a fifth and paradoxical element within this framework. Rather than neutralising folk hero narratives, platform interventions—flagging, restricting, or removing posts—are frequently reinterpreted by users as evidence of institutional suppression, functioning as an accelerant rather than a brake on anti-elite sentiment. In this sense, moderation becomes constitutive of the very narrative it seeks to contain.

All of these forces operate within the digital platform environment, particularly Reddit, whose affordances (anonymity, upvotes, and meme circulation) facilitate the creation and spread of these reframings. At a certain threshold of virality, platforms like Reddit may intervene through content moderation, flagging, restricting, or removing posts. However, this often reinforces the populist narrative that powerful institutions (corporations, the state, or even Big Tech) suppress dissent. This leads to a feedback loop: content moderation interventions risk being framed as institutional suppression of dissent, thereby deepening anti-elite sentiment and amplifying the folk status of the figure being moderated.

This cyclical process, grievance, framing, validation, suppression, and myth-making, demonstrates how digital media do not just reflect public sentiment but actively participate in reshaping the boundaries of morality, justice, and heroism in the post-truth age. The Luigi Mangione phenomenon is therefore not an anomaly, but a crystallisation of broader sociotechnical and ideological shifts, where platforms become moral battlegrounds, and memes become modern-day legends.

### 2.6 Research questions

The theoretical framework outlined above—integrating digital populism, moral disengagement, spectacle culture, and content moderation—provides the conceptual scaffolding for the following research questions, which this study seeks to address: (1) How do Reddit users frame and reinterpret the alleged murder of a healthcare CEO by Luigi Mangione? (2) What thematic narratives emerge in Reddit discourse around Mangione (e.g., heroism, injustice, vengeance)? (3) How do these online narratives reflect broader social sentiments about institutions and power (such as class resentment or distrust in justice systems)? These questions oriented our coding towards understanding not just *what* was being said, but *why*, what grievances or values underpinned the surprising support for an accused killer.

## 3 Methods

This study employed a qualitative research design to examine how anonymous online communities reframe morally ambiguous acts into symbolic narratives of justice.

### 3.1 Research participants and sampling

Data were collected between April and June 2025, covering Reddit threads posted between December 2024 and April 2025—the period of most intense public discourse around the case. Rather than automated scraping, a structured manual search was carried out using keywords such as “Luigi Mangione,” “UnitedHealth CEO,” “healthcare murder,” “Brian Thompson,” and “UHC killing.” Threads were identified from an initial set of seven subreddits selected for thematic relevance and ideological diversity: r/politics, r/popculture, r/AskReddit, r/technology, r/Fauxmoi, r/antiwork, and r/WorkReform.

Inclusion criteria for threads required (a) direct reference to the Mangione case, (b) high comment volume, and (c) visible evidence of sentiment polarisation or narrative reframing. Within selected threads, comments were sorted by “Top” to capture the most upvoted and visible posts. Following Massanari (2015) and Proferes et al. (2021), upvotes are treated here as indicators of discursive resonance rather than community consensus—reflecting content that achieved high visibility and shaped collective meaning-making, not the distribution of opinion across Reddit as a whole.

Reddit’s ranking logics privilege content that generates strong affective responses within a given community at a given moment, and highly upvoted posts may reflect the preferences of the most active or vocal users rather than the subreddit’s broader membership. Accordingly, we treat top-ranked comments not as representative of community consensus but as indicative of discursive resonance—content that achieved high visibility and therefore had the greatest potential to shape and reinforce collective meaning-making within the thread. This distinction is analytically important: the study’s claims are about the discourse that achieved platform visibility, not about the distribution of opinion across Reddit’s user base as a whole.

The final dataset comprised 6,466 comments across 38 threads spanning 14 subreddits (see Table 1). As manual exploration proceeded, threads meeting the inclusion criteria were identified beyond the initial seven subreddits through Reddit’s organic cross-posting culture, expanding the dataset to include communities such as r/pics, r/SimpsonsShitposting, r/FluentInFinance, r/MurderedByWords, r/InterestingAsF#*k, r/WhitePeopleTwitter, r/BlackPeopleTwitter, r/LinkedInLunatics, r/toptalent, and r/AdviceAnimals. Their inclusion demonstrates that folk hero narratives extended well beyond activist spaces into mainstream, humour, finance, and pop culture communities. Collectively, these threads generated over 3.3 million upvotes. Subreddit sizes ranged from under 300,000 to over 33 million members.

**Table 1:** Overview of Reddit threads analysed in the study.

Number	Page	Rank by size	Members	Total comments	Total up votes on the page
1	Oddlyspecific	Top 1%	3.7 M	195	30,628
2	Technology	39	20 M	101	96,103
3	Simpsons Shitposting		270 K	174	32,237
4	Advice Animals	Top 1%	9.9 M	194	40,132
5	WorkReform	Top 1%	759 K	182	29,777
6	FluentInFinance	Top 1%	567 K	192	21,251
7	Pics	12	33 M	163	194,722
8	Technology	39	20 M	191	123,991
9	Pics	12	33 M	181	197,508
10	Politics	Top 1%	8.9 M	182	151,093
11	MurderedByWords		3.3 M	195	42,412
12	Technology	39	20 M	195	74,923
13	MurderedByWords		3.3 M	186	206,699
14	LinkedInLunatics	Top 1%	821 K	197	17,302
15	WorkReform	Top 1%	759 K	196	35,203
16	Antiwork	Top 1%	2.9 M	191	18,471
17	BlackPeopleTwitter		6.1 M	101	143,600
18	Antiwork	Top 1%	2.9 M	189	110,797
19	Fauxmoi	Top 1%	6.4 M	125	87,602
20	WorkReform	Top 1%	759 K	194	42,605
21	Pics	12	33 M	176	181,504
22	Antiwork	Top 1%	2.9 M	189	100,953
23	WorkReform	Top 1%	759 K	188	13,169
24	Pics	12	33 M	171	249,960
25	WhitePeopleTwitter	Top 1%	3.1 M	118	27,907
26	Technology	39	20 M	179	118,091
27	Toptalent	Top 1%	3 M	100	125,055
28	WorkReform	Top 1%	759 K	185	17,086
29	Interesting As Fuck	Top 1%	15 M	183	259,344
30	WhitePeopleTwitter	Top 1%	3.1 M	116	122,615
31	WhitePeopleTwitter	Top 1%	3.1 M	174	38,308
32	Antiwork	Top 1%	2.9 M	188	60,338
33	WhitePeopleTwitter	Top 1%	3.1 M	144	64,907
34	WorkReform	Top 1%	759 K	185	133,379
35	WhitePeopleTwitter	Top 1%	3.1 M	195	64,398
36	WorkReform	Top 1%	759 K	73	8,911
37	Simpsons Shitposting		270 K	193	27,617
38	WorkReform	Top 1%	759 K	185	36,717

Each comment was logged with subreddit name, date, upvote count, and full text. The dataset was reviewed by a second researcher (the co-author) who independently assessed metadata accuracy, thematic relevance, and contextual fit. Disagreements were resolved through discussion, with ambiguous cases excluded. Trustworthiness was established through procedural documentation, transparent inclusion criteria, and iterative consensus, consistent with best practise in qualitative research (Braun and Clarke, 2013, 2021; Lincoln and Guba, 1985).

### 3.2 Data analysis

Data analysis followed the six phases of reflexive thematic analysis (Braun and Clarke, 2006, 2021). In Phase 1, both researchers independently read through the full dataset multiple times to achieve deep familiarity with the material. In Phase 2, initial codes were generated by systematically identifying features of the data relevant to the research questions including emotional outpour, and rhetorical moves such as moral justification, institutional critique, and aesthetic framing. In Phase 3, codes were sorted into candidate themes by grouping those that shared conceptual coherence. Phase 4 involved reviewing and refining these candidate themes against the full dataset to ensure they were internally consistent and meaningfully distinct from one another. In Phase 5, themes were defined and named, with each assigned a clear analytic focus. Phase 6 involved the production of the final thematic narrative, selecting representative extracts and constructing the analysis presented in the Findings section.

Thematic dominance was established through interpretive rather than frequency-based criteria, in keeping with the epistemological commitments of reflexive thematic analysis. Braun and Clarke (2021, p. 102) explicitly caution against equating prevalence with significance in RTA, noting that word frequency counts are more appropriate to content analysis or corpus linguistics than to meaning-focused interpretive work. Accordingly, the researchers considered a theme dominant when it met three criteria: it recurred meaningfully across multiple subreddits rather than being confined to a single community; it carried strong community endorsement as evidenced by high upvote counts on representative comments; and it achieved convergence between both coders during the independent review phase, with any disagreements resolved through discussion. These criteria ensured that the identified themes reflected patterns of collective meaning-making with broad discursive reach.

### 3.3 Ethical considerations for Reddit data analysis

This study involved secondary analysis of publicly available Reddit comments and did not require formal Institutional Review Board (IRB) approval. According to the Department of Health, Republic of South Africa (2015, Section 1.1.8), research relying exclusively on publicly available digital content—where no direct interaction with participants occurs—is typically exempt from formal ethics review (Department of Health, Republic of South Africa, 2015). This position is consistent with broader guidance on internet research ethics, which recognises public online spaces as contexts where formal review requirements are generally not triggered (Fiesler et al., 2024; Markham and Buchanan, 2012; Proferes et al., 2021). This study meets all relevant exemption criteria: data were publicly accessible without authentication; no recruitment or contact with users occurred; Reddit’s platform architecture and terms of service establish that public forum posts carry no reasonable expectation of privacy; and no usernames, identifiers, or permalinks were collected or reported.

Notwithstanding the exemption, established ethical principles for social media research were followed throughout (Fiesler et al., 2024; Proferes et al., 2021). All comments were anonymised prior to analysis, findings are presented without individual attribution, and verbatim quotes are contextualised to minimise the risk of reverse-identification through search engines (Reagle, 2022). The dataset is stored securely; should verification be required, it can be made available via screen-sharing upon reasonable request.

## 4 Findings

### 4.1 Thematic analysis of Reddit discourse

To illuminate how Reddit users have been discussing the Luigi Mangione case, we identified five major themes in the discourse. Each theme is illustrated with representative Reddit comments (paraphrased). These themes capture the spectrum of public sentiment, from glorification of Mangione as an anti-establishment folk hero to cynical distrust in institutions, narratives of systemic injustice, forms of digital protest, and the aestheticisation of the accused in online culture.

#### 4.1.1 Theme 1: Folk hero glorification and moral inversion

A dominant theme in the Reddit discourse is the portrayal of Luigi Mangione as a folk hero, an emblem of righteous defiance against perceived systemic injustice. Despite being an accused murderer, Mangione is elevated in the eyes of many commenters to a status not only of moral innocence but of near-sacred virtue. This moral inversion transforms him from a criminal suspect into a symbolic saviour, reflecting what Bandura (1999) refers to as *moral disengagement*: the re-framing of violent acts as morally justifiable when they serve a perceived greater good.

**Figure 3:**
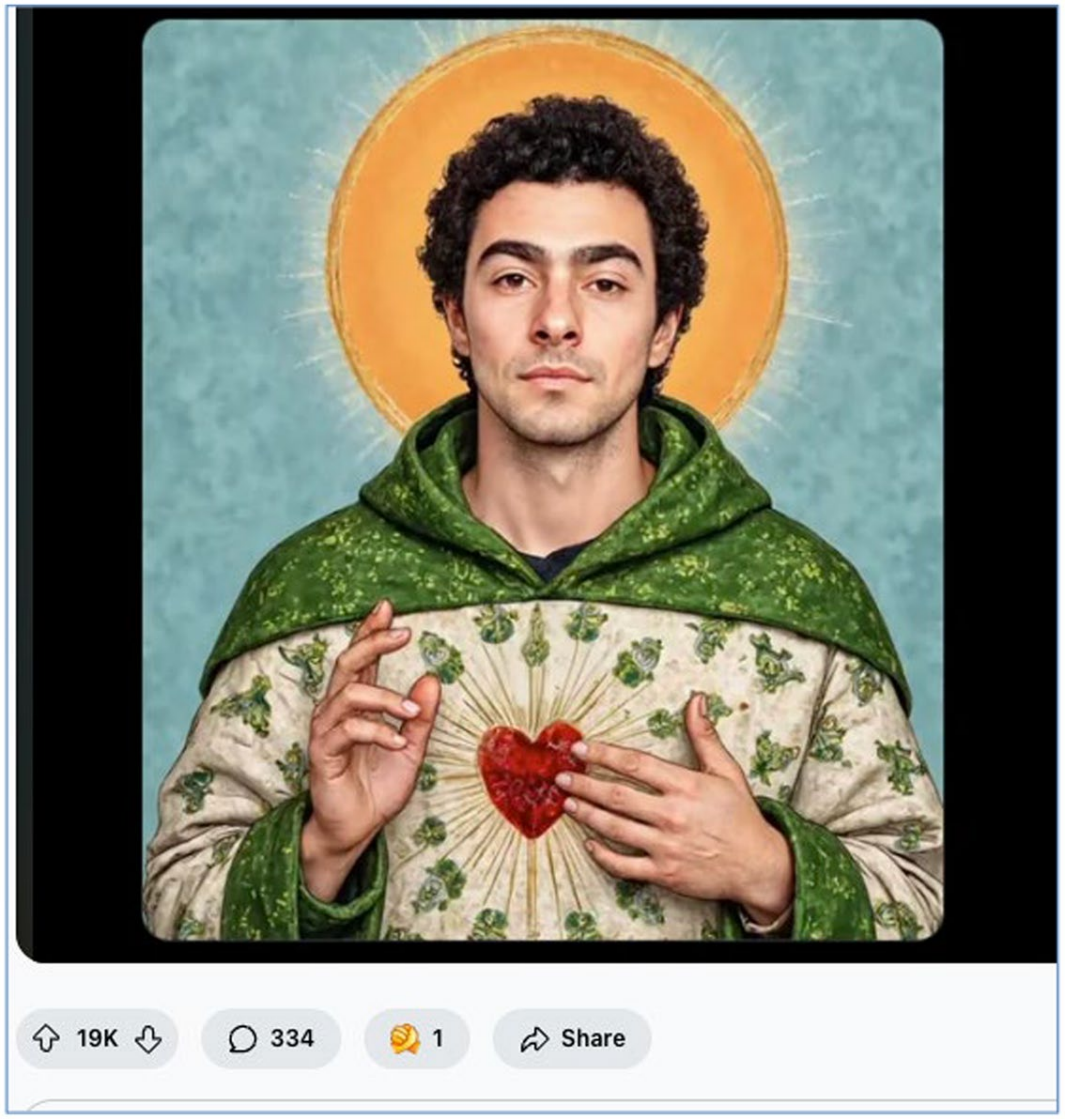
“Saint Luigi” meme as a symbol of digital folk heroism (May 2025, image retrieved from Reddit). This AI-generated meme-style image of Luigi Mangione, designed in the likeness of a Catholic saint, was widely circulated on Reddit as part of the “Saint Luigi” discourse. The sacred imagery and aesthetic framing reflect how online communities mythologised Mangione as a martyr or anti-establishment saviour. The post received over 19,000 upvotes, illustrating the emotional resonance of the image. Image retrieved from Reddit.

Across multiple subreddits, users glorified Mangione as a “*man of the people*,” casting him as someone who stood up to entrenched elites on behalf of ordinary citizens. Redditors invoked spiritual iconography, with one commenter remarking: “*Day by day, he looks more like a saint. May Saint Luigi bless us all.*” The discourse repeatedly suggested that Mangione’s alleged actions were not only understandable but morally virtuous. As one user wrote, “*It’s not a crime when it’s done to save the country*.” Here, violence is cast as redemptive, a necessary act in service of the collective. This rhetorical shift from condemnation to canonisation reflects a deeper social yearning for justice, particularly among those disillusioned by institutional authority. Figure 3 illustrates how this sentiment extended into visual culture.

#### 4.1.2 Theme 2: Distrust in institutions and “folk justice”

A recurring thread in the Reddit discourse is a deep and visceral distrust in formal institutions, particularly the judiciary, law enforcement, and mainstream media. Across the dataset, users consistently portray these bodies not as neutral arbiters of truth or justice, but as biassed instruments of elite control. This sentiment gives rise to an alternative logic of “folk justice”: a populist belief that justice must be reclaimed or re-enacted by ordinary people when the state fails to deliver it fairly.

Commenters argued that Luigi Mangione could never receive a fair trial, due to what they perceived as prejudicial media framing and political motivations. One user remarked, “*He will not get a fair trial, the media’s already painted him as guilty,*” while another lamented, “*You cannot even find an unbiased jury after the way he’s been portrayed. The whole case should be thrown out.*” These statements reflect a growing belief in the irreparability of procedural justice when powerful interests are involved.

Others identified hypocrisy and incoherence in state discourse, especially regarding capital punishment. As one commenter put it: “*It’s wild—some people scream about government corruption, then in the next breath demand the government execute more people. Which is it?*” This contradiction points to a deeper crisis of legitimacy, where state violence is viewed not as justice but as a means of suppressing dissent. Another user warned: “*If they go through with the death penalty, they are only going to make him a martyr. This all feels like a tactic to scare people who might follow in his footsteps.*”

**Figure 4:**
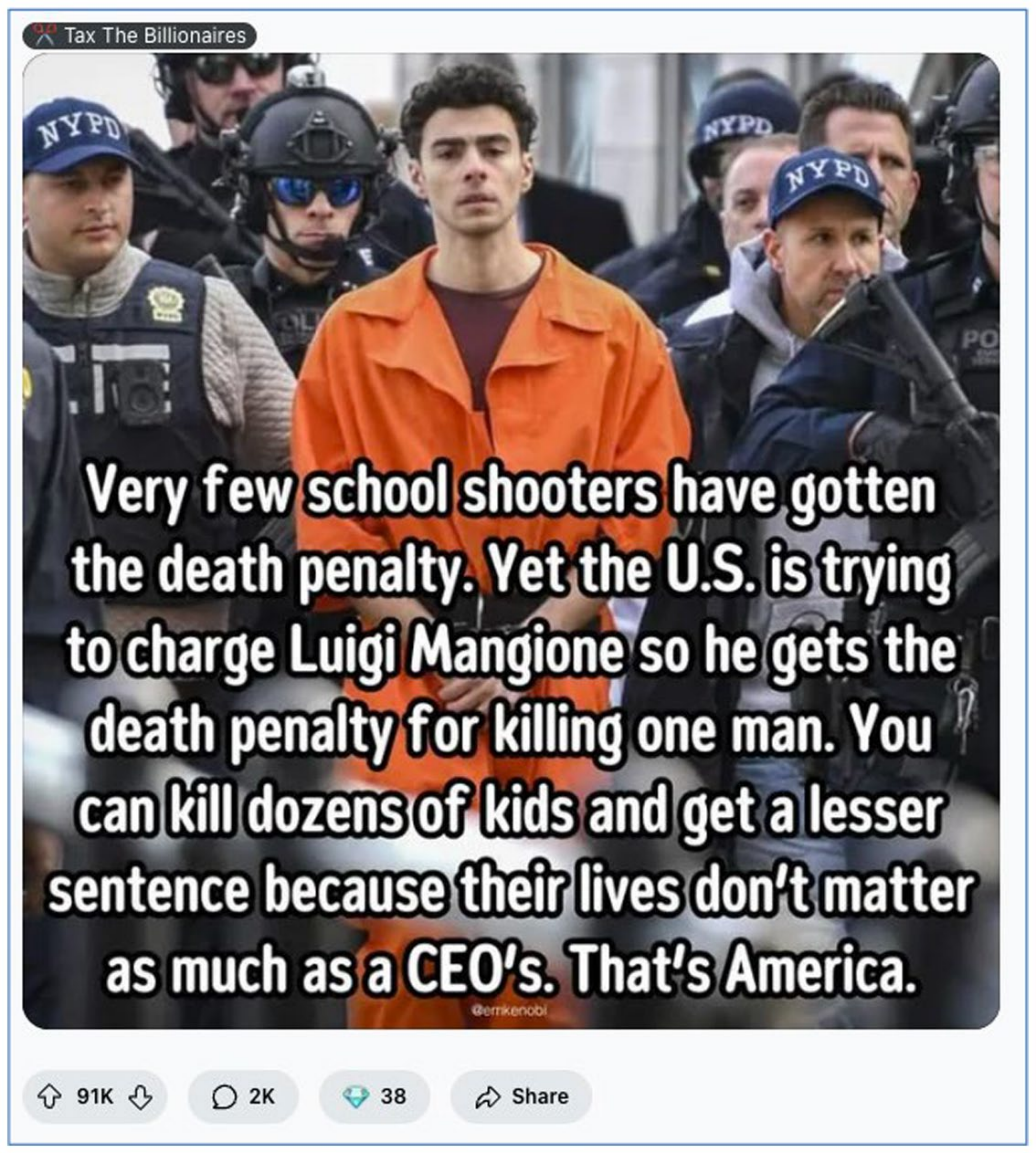
Comparative justice meme critiquing the U. S. legal system (March 2025, image retrieved from Reddit). This meme from r/*Workreform* juxtaposes Luigi Mangione’s prosecution with the treatment of school shooters to highlight perceived systemic bias in the American justice system. The image, widely shared on Reddit, argues that corporate elites receive disproportionate legal protection compared to ordinary citizens or children. With over 91,000 upvotes and 2,000 comments, this meme encapsulates the sentiment of institutional distrust and class-based moral outrage.

Collectively, these sentiments articulate a form of digital populism in which the justice system is imagined not as flawed but fundamentally illegitimate. In this worldview, justice must be crowdsourced, expressed through memes, upvotes, and shared outrage rather than court verdicts. The trial of Mangione becomes less about legal facts and more about symbolic warfare between the elite and “the people.” This dynamic is vividly illustrated in Figure 4, a meme that crystallises institutional distrust through a provocative visual comparison.

#### 4.1.3 Theme 3: Systemic injustice, moral outrage and catharsis

A third thematic pattern emerging from the Reddit data is the fusion of moral outrage with cathartic justification, grounded in collective experiences of systemic injustice, particularly within the U. S. healthcare system. In this narrative, Luigi Mangione becomes a symbol of karmic retaliation against a profit-driven healthcare regime that has inflicted suffering on thousands. The alleged act of violence is thus reinterpreted not as a moral transgression but as an emotionally legible—and even righteous—response to prolonged structural harm.

Numerous commenters described firsthand or witnessed suffering related to healthcare denial, economic abandonment, and bureaucratic cruelty. As one user wrote, “*What about all the painful deaths of people denied life-saving care by insurance companies*?” Others juxtaposed corporate impunity with individual punishment, highlighting the asymmetry between the lives lost under systemic negligence and the state’s swift response to retaliatory acts. Referencing the arrest of a woman who mentioned the phrase *“Delay, Deny, Depose,”* a term associated with insurance denial tactics, one commenter observed: “*She uttered three words and got arrested. Meanwhile, insurance companies follow that motto daily and call it ‘business.*”

This theme also reveals a moral disengagement mechanism (Bandura, 1999), whereby conventional ethics are suspended in contexts where the system itself is perceived as corrupt. “*I’m not saying I endorse violence*,” one user clarified, “*I’m done pretending to feel sad about corporate executives. They showed us zero empathy. Why should we offer them any*?” Sympathy is withheld not as a lapse in compassion, but as an act of moral parity, a refusal to grieve for those who profit from others’ pain.

**Figure 5:**
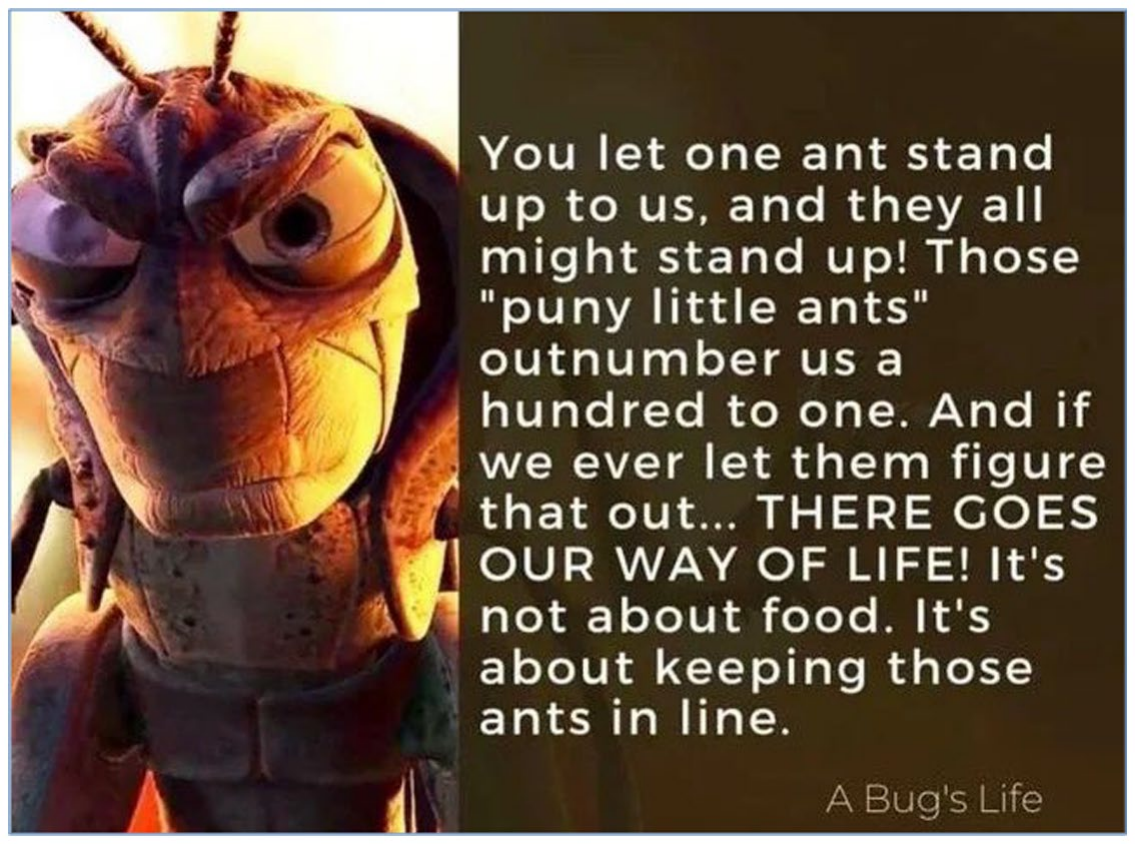
“A Bug’s Life” meme as a parable of class power and suppression (March 2025, image retrieved from Reddit). This meme, referencing the film *A Bug’s Life*, was shared in a r/*FreeLuigi* discussing the Luigi Mangione case and received over 1,700 upvotes. It features the villain Hopper warning: “You let one ant stand up to us, and they all might stand up! … It’s not about food. It’s about keeping those ants in line.” Redditors interpreted this quote as a stark metaphor for elite fear of collective resistance, framing Mangione’s harsh prosecution not as a pursuit of justice, but as an attempt to deter mass defiance. The post reinforces a recurring narrative in Reddit discourse: that institutional responses are more concerned with preserving hierarchical control than with morality.

Underlying these expressions is a clear diagnosis: the true conflict is class warfare. As one user succinctly put it, “*Notice who he fears, it’s not immigrants or minorities. It’s the poor. That tells you everything about who threatens the system*.” This class warfare framing is not incidental to Reddit’s discursive culture but structurally embedded within it. Massanari (2015) has long noted that Reddit’s participatory economy is shaped by the cultural preferences of a predominantly white, male, and technically literate demographic—what she terms “privileged geek masculinities”—whose anti-establishment rhetoric frequently coexists with blind spots around race, gender, and structural privilege. Ging (2019) similarly identifies how anti-elite narratives in male-dominated online spaces can simultaneously critique economic inequality while reproducing other forms of social hierarchy. The class warfare diagnosis that emerges in the Mangione discourse should therefore be read as a culturally situated framing—one that reflects the specific political economy of Reddit’s user base rather than a universal or ideologically neutral diagnosis of systemic inequality. Others echoed this refrain, suggesting that Mangione’s prosecution is not about justice but about suppressing dissent: a warning shot to anyone who dares to challenge entrenched economic hierarchies. Figure 5 captures this logic of power and resistance through the allegorical lens of animation.

#### 4.1.4 Theme 4: Digital protest and anti-corporate activism

A defining element of the Luigi Mangione discourse is the transformation of public sentiment into collective digital resistance. In this theme, Redditors move beyond rhetorical support and actively engage in grassroots activism, both online and offline, aimed at challenging dominant corporate narratives. These acts, ranging from *review-bombing* fast-food chains to celebrating defiant graffiti, reflect an emerging form of digital populism: decentralised, affect-driven, and rooted in shared outrage against institutional power.

One notable example was the coordinated *one-star review campaign* targeting a McDonald’s outlet allegedly linked to Mangione’s arrest. Although users acknowledged the performative nature of the act, “*No one checks McDonald’s reviews anyway*,” the action was celebrated as symbolic defiance. It served as both catharsis and critique, highlighting how everyday digital platforms can become tools of resistance. Similarly, mentions of street art and graffiti depicting Mangione as a rebel or martyr were met with admiration, seen as subversive re-inscriptions of public space.

Another layer of protest emerged in critiques of selective moderation and executive secrecy. Users noted that pro-Mangione posts were swiftly removed across platforms, interpreting this as proof of corporate hypersensitivity: “*Funny how fast these platforms enforce content rules when their sponsors feel threatened*.” Others observed that executives began appearing less in public or deleted social media traces, reading this withdrawal as an admission of guilt or, at minimum, of reputational vulnerability. These discussions reflect a deepening suspicion towards digital governance, where moderation is seen not as neutral enforcement but as corporate self-protection.

**Figure 6:**
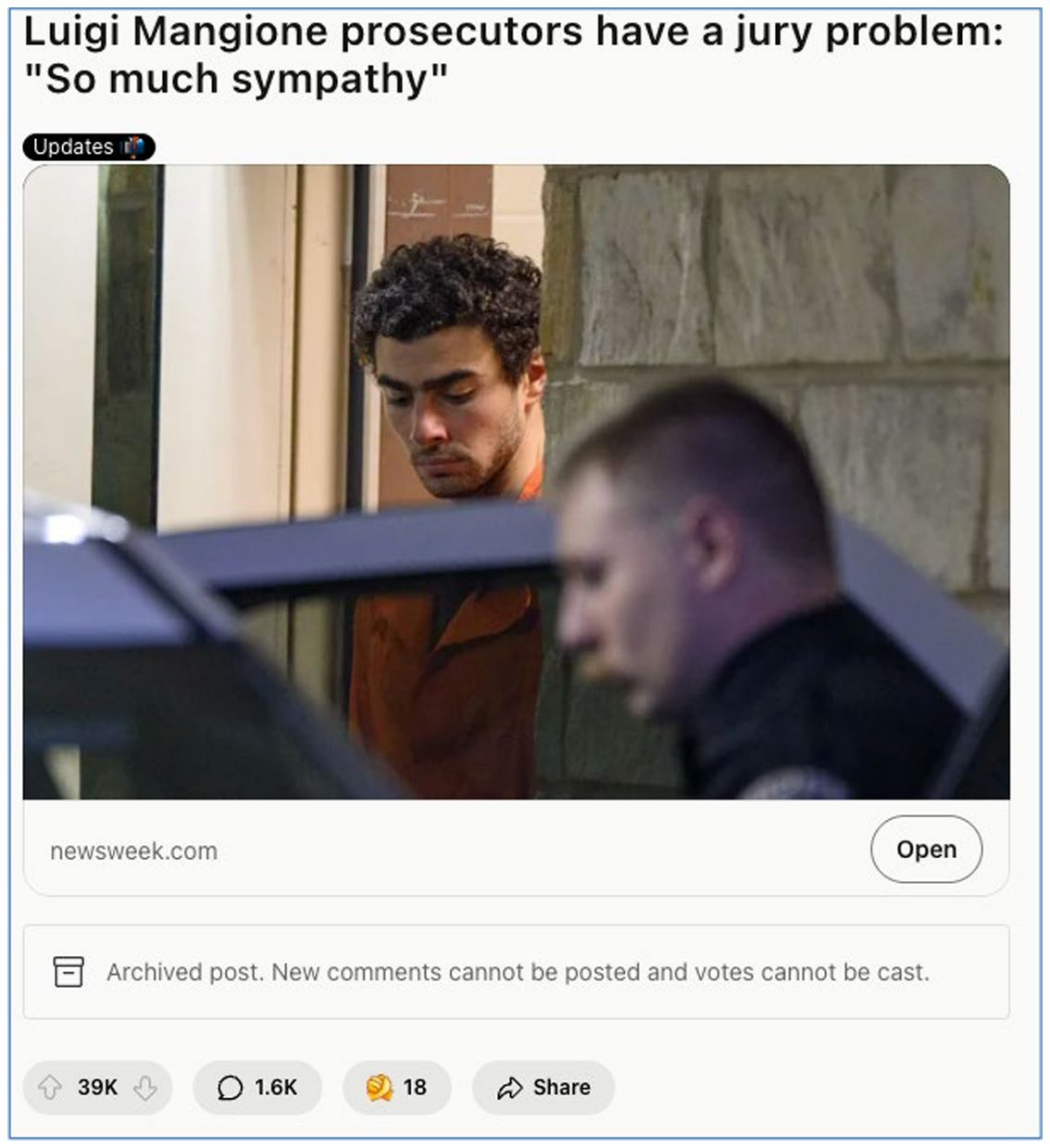
The jury of the people: Sympathy, distrust, and class disillusionment (March 2025, image retrieved from Reddit). This post from r/antiwork (39 K upvotes) shows how Redditors viewed the legal proceedings as biassed and class-driven. Highly upvoted comments criticised the terrorism charge as baseless and the justice system as favouring the elite. Many expressed doubt that an impartial jury could be found, given widespread resentment towards health insurers (O’Driscoll, 2024).

Beyond Reddit, activism spread into broader forums such as r/antiwork, where users reframed the trial as class warfare masquerading as justice. Highly upvoted comments questioned whether a fair jury could ever be assembled, given the widespread economic pain inflicted by private healthcare. “*Good luck finding twelve Americans (as jury) who have not been screwed by an insurance company*,” one user quipped, highlighting the impossibility of neutrality in a system many feel is structurally unjust (see Figure 6). These forms of protest and critique signal a broader evolution of digital space—from a site of commentary to a theatre of resistance and populist organising.

#### 4.1.5 Theme 5: Spectacle and the aesthetic of resistance

Among the most distinctive phenomena observed in the Reddit commentary on Luigi Mangione is the convergence of spectacle, aesthetics, and irony-laced adulation. Especially prominent in pop culture–oriented subreddits such as *r/Fauxmoi*, users transform Mangione from a criminal defendant into a celebrity icon. His courtroom appearances are dissected as if they were fashion statements; his composure is admired like that of a seasoned actor on a press tour. The resulting discourse marks a striking fusion of digital fan culture with political subversion, an emergent aesthetic of resistance where glamour undermines state power.

Users frequently describe Mangione with language more appropriate to red carpet events than court proceedings. One quipped, *“So this is what Beatlemania must have felt like,”* while another mock-confessed, *“That second photo… has me contemplating a revolution.”* His outfits, particularly a dark green sweater, became part of a meme lexicon: *“Dark green suits him perfectly… of course it’s Luigi’s signature colour.”* The fashion-forward framing is not simply superficial. Rather, it deflates the gravitas of the trial, shifting the narrative from one of institutional justice to one of emotional and visual alignment with the accused.

This aestheticisation draws on the logic of *spectacle culture* (Debord, 1967), where *public trials become performance stages*, and individuals on trial are consumed as media content rather than legal subjects. The courtroom is no longer a site of deliberative democracy but a highly stylised theatre in which affect, beauty, and meme potential govern perception. This framework allows users to ironically reclaim power, turning their focus to Mangione’s poise, looks, and fashion to destabilise the authority of legal procedure.

**Figure 7:**
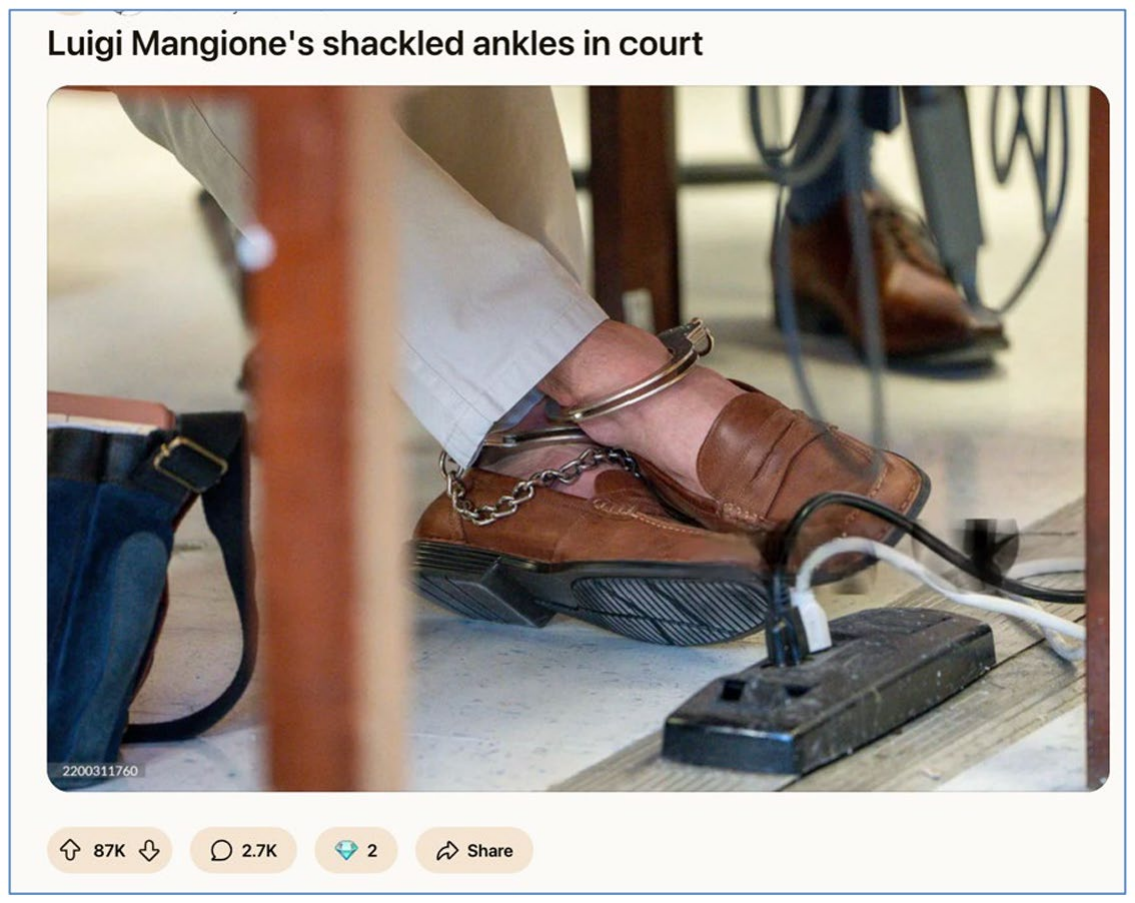
The aesthetics of confinement: Mangione’s shackles as spectacle (March 2025, image retrieved from Reddit). This widely shared image from r/pics (87 K upvotes) focuses on Luigi Mangione’s shackled ankles during his court appearance. Rather than eliciting fear or condemnation, Redditors responded with commentary that emphasised his calm posture, clean shoes, and quiet dignity. In line with s*pectacle culture* (Debord, 1967), the courtroom becomes a stage, and Mangione’s body becomes a site of narrative contestation—where the state sees restraint, the crowd sees martyrdom. This visual fed into his mythologization as “Saint Luigi,” further detaching public sentiment from the actual violence involved.

Importantly, the celebration of Mangione’s appearance operates as a coded form of resistance. It does not deny the violence of the act but *redirects attention towards the inequities of the system,* suggesting that the real spectacle is not the defendant, but the institutions that presume to judge him. In praising Mangione’s aesthetic dignity under literal shackles, users invert the power dynamic: the state may hold the chains, but the crowd controls the narrative. This inversion is best captured in Figure 7, which visually encapsulates the dynamics of visual resistance and semiotic reframing.

## 5 Discussion

The Reddit discourse analysed here offers a vivid illustration of how digital platforms function as arenas for moral reframing, class critique, and grassroots myth-making. Across over 6,400 comments, users transformed the Luigi Mangione case from a criminal proceeding into a broader populist allegory, one centred on resentment towards corporate power, media complicity, and elite impunity.

This dynamic is not unique to the Mangione case. Across different cultural contexts, digital publics have consistently constructed folk hero narratives around figures perceived to be striking back against institutional failure. Albarran-Torres and Goggin (2023) document how Joaquín “El Chapo” Guzmán was mythologised as a “peasant Robin Hood” on Mexican social media, his criminal identity subordinated to a populist narrative of humble origins and resistance to state power. Similarly, Castle and Parsons (2019) show how the Soldiers of Odin Norge reframed their vigilante identity through Viking archetypes, positioning themselves as heroic community protectors rather than extremists. Klein (2015) further demonstrates that global press coverage of Anonymous oscillated between “vigilante hero” and “global threat,” underscoring how the legitimacy of transgressive figures is always contested and contingent on perceived social impact. What these cases share with the Mangione phenomenon is the centrality of institutional distrust as the precondition for folk heroisation—when formal systems are seen to have failed, digital publics construct their own moral authorities.

This interpretive shift reflects a wider political malaise. Pew Research Center (2024) reports only *22% of Americans* express consistent trust in the federal government, a figure that drops further among working-class and white Americans. Trust has steadily eroded since the 1960s, punctuated by scandals, economic dislocation, and a perception that institutions no longer represent “ordinary people.” In this context, Reddit’s turn towards folk justice and moral inversion is not anomalous, it is symptomatic. While the institutional and economic data cited here are US-specific, the analytical claims speak to broader patterns of digital populism and institutional distrust documented across multiple national contexts (Gerbaudo, 2018; Hameleers, 2020).

**Figure 8:**
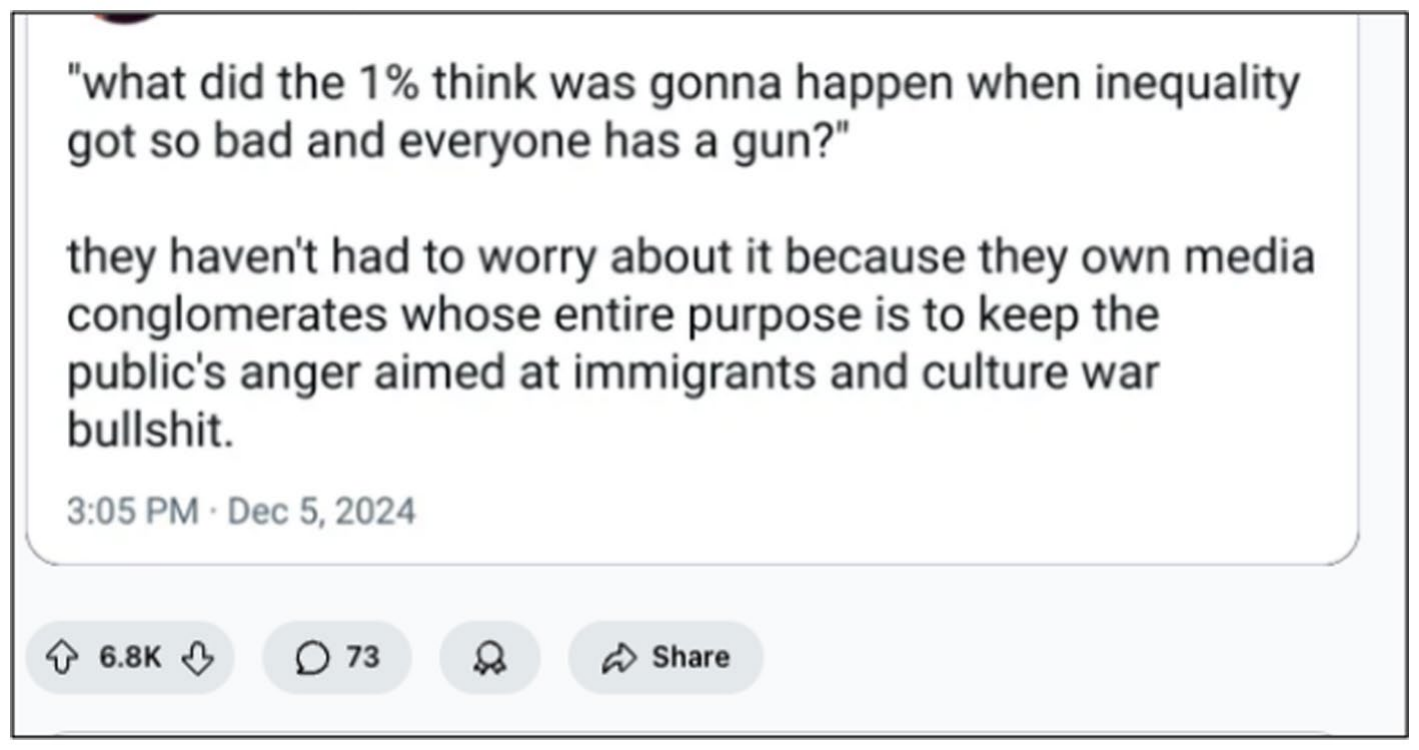
The guns of inequality: Populist rage and the failure of distraction politics (February 2025, image retrieved from Reddit). This top-voted Reddit post from r/WorkReform (6.8 K upvotes) expresses the belief that extreme inequality, coupled with mass access to firearms, inevitably leads to violent unrest. The comment critiques elite-owned media for redirecting public frustration towards scapegoats—immigrants, minorities, or culture wars—rather than addressing economic injustice. As discussed in the Digital Populism section of our framework, such narratives reveal how users perceive the Mangione case not as isolated violence but as the breaking point in a long-ignored class conflict.

Economic inequality further amplifies this logic. The *top 1% of Americans now earn more than twice as much as the bottom 50% combined* (Saez and Zucman, 2019), a disparity mirrored in Redditors’ repeated refrains about the CEO “deserving” what happened because of healthcare injustices. As shown in Figure 8, users linked systemic inequality to inevitable unrest, critiquing the media for redirecting public anger towards scapegoats like immigrants and minorities. Here, violence was not excused but reframed as karmic backlash against unchecked corporate cruelty.

The mechanisms through which this moral reframing operates are consistent with findings from comparative vigilantism research. Jönsson (2024) identifies that support for vigilantism characteristically stems from a perception that the criminal justice system is dysfunctional or corrupt, allowing citizens to justify extra-legal force as a necessary corrective. In the Mangione case, this manifested through Bandura’s (Bandura, 1999) moral disengagement mechanisms—euphemistic labelling, advantageous comparison, and dehumanisation of the victim—which allowed users to reframe a killing as karmic justice. Cheong and Gong (2010) document a comparable dynamic in China, where “human flesh search” campaigns were framed as tools for restoring public morality when the state failed to act. Huang (2023) cautions, however, that such moral disengagement can slide into harassment and exclusion when nationalist or misogynist currents are present—a reminder that the same cognitive mechanisms that produce folk heroisation can also produce mob justice. The Mangione case sits at the more sympathetic end of this spectrum, but the underlying architecture of moral justification is structurally similar across contexts.

**Figure 9:**
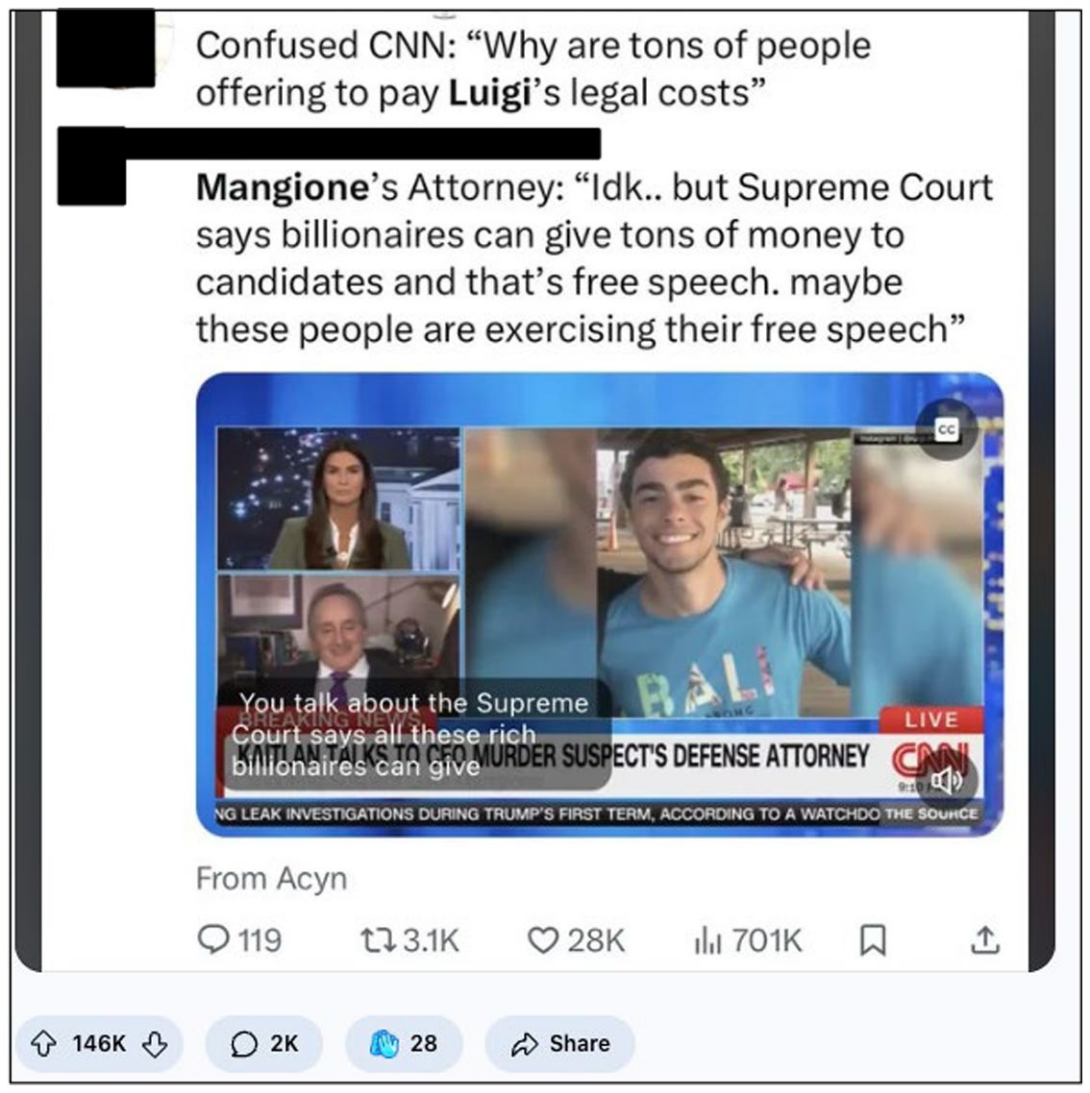
Crowdfunding as protest: Free speech, legal defence, and popular legitimacy (August 2025, image retrieved from Reddit). This post from r/MurderedByWords (146 K upvotes) captures how Mangione’s legal defence became a symbol of grassroots resistance. The attorney’s sarcastic reference to campaign finance law reframes public donations as an act of democratic free speech, implying that if billionaires can legally fund politicians, ordinary citizens should be able to fund a folk hero. This moment signals how users interpreted financial support not only as solidarity, but as a political expression challenging elite norms.

This sentiment extended beyond symbolic endorsement into material action. Supporters reframed GoFundMe donations as democratic resistance. As one high-engagement post sarcastically noted, “if billionaires can fund politicians, we can fund a folk hero” (Figure 9). This act of crowdfunding was not merely financial—it was symbolic, expressing political legitimacy from below.

The role of platform affordances in shaping these narratives warrants specific attention. Trottier (2017) identifies the “weaponisation of visibility” as a defining feature of digital vigilantism, where coordinated public attention produces consequences that transcend geographical boundaries. Demelius and Yoshida (2025) further show how the attention economy of platforms like YouTube incentivises vigilantes to prioritise entertainment and viewership, blurring the boundary between justice-seeking and spectacle. Gerbaudo (2018) documents how algorithmic filter bubbles allow groups to construct moral solidarity away from mainstream scrutiny—a dynamic clearly visible in Reddit’s upvote-driven communities. As Marantz (2019) observed, online platforms increasingly serve as counter-publics where dominant media narratives are challenged, reassembled, and meme-ified. The aestheticisation of Mangione (his sweater, shackles, stoicism) taps into this dynamic. This is a tactic of legitimacy laundering, where humour, spectacle, and moral outrage converge to recode state violence and elite privilege as the true aberrations. Justice, in this arena, is not what courts declare, it is what the crowd affirms.

In short, the Mangione discourse encapsulates a deeper shift in public reasoning. When crowdfunding is protest, when shackles are rebranded as fashion, and when violence is sanctified through memes, it suggests not merely a breakdown of institutional trust, but the emergence of a new cultural logic, where justice is judged not by legality, but by perceived righteousness. As the comparative literature demonstrates, this logic is an emergent property of digital platforms wherever institutional trust has eroded and affordances for anonymous, affect-driven discourse exist.

## 6 Conclusion

The Reddit discourse around Luigi Mangione’s case highlights a profound crisis of legitimacy in contemporary online communities. Across five themes, users collectively inverted conventional moral judgement. They valorised an accused killer while withholding sympathy from his victim. This was driven by grievances around corporate injustice, economic inequality, and institutional failure—grievances so pervasive that they overrode ordinary norms of condemnation. On Reddit, these frustrations were not merely expressed but organised—through upvotes, memes, and collective storytelling—into a coherent populist narrative.

The findings carry several implications. First, the Mangione case demonstrates that social media can rapidly aggregate and amplify counter-narratives. What might have been a fringe opinion quickly became mainstream sentiment across large subreddits, indicating a level of societal anger that warrants serious attention. Policymakers and institutional leaders who dismiss such digital signals risk misreading the depth of public disillusionment—the pro-Mangione movement, however irreverent at times, stems from genuine pain and perceived injustice.

Second, moral discourse on social media is rarely straightforward. In this case, humour, satire, and spectacle sat alongside genuine outrage—and the two were often inseparable. Researchers and communicators should recognise internet culture not as a distraction from serious discourse, but as the primary medium through which it now frequently travels.

Third, the Mangione case reveals the double-edged nature of digital counter-publics. Reddit’s semi-anonymous, vote-driven architecture can amplify marginalised voices and hold institutions to account—but the same affordances that enable democratic expression can normalise vengeance and mob celebration of violence. As Huang (2023) and Trottier (2017) caution, moral disengagement can slide from political critique into targeted harassment, a boundary digital platforms are structurally ill-equipped to enforce.

Ultimately, the Redditors rallying around Luigi Mangione were voicing a collective verdict on the state of things: when people feel systemically abandoned, they seek justice by alternative means. The online lionisation of an accused killer is not an endorsement of violence—it is a signal of how far institutional legitimacy has eroded.

**Figure 10:**
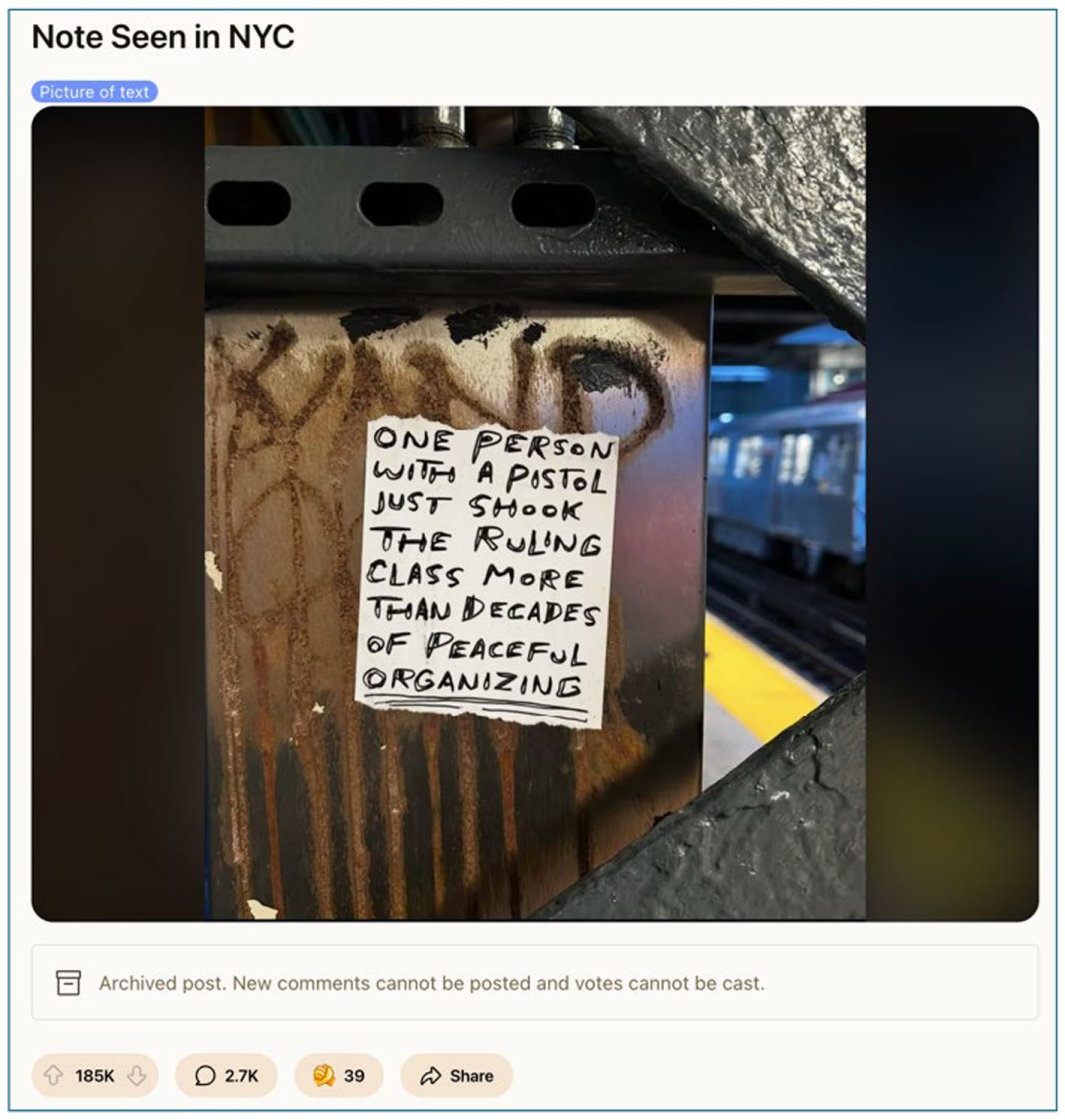
Subway graffiti as class commentary: The power of a single act (March 2025, image retrieved from Reddit). This subway note, shared on r/pics (185 K upvotes), proclaims that “one person with a pistol just shook the ruling class more than decades of peaceful organising.” It encapsulates the radical sentiment circulating online post-Mangione. As a piece of public ephemera, it ties directly to themes of d*igital populism* and s*pectacle culture*, expressing the belief that direct, even violent, disruption garners more impact than conventional advocacy. The post’s virality reflects a broader cultural reckoning with power, legitimacy, and the limits of sanctioned dissent.

The key finding of this study is that digital discourse does not occur in a vacuum; it is deeply intertwined with real-world grievances (see Figure 10). In the figure of “Saint Luigi,” we see a society reaching for accountability in a world perceived to lack it—one meme, upvote, and folk myth at a time.

## 7 Limitations and recommendations

Several limitations should be noted. First, the analysis relies exclusively on publicly available Reddit posts. While anonymity fosters uninhibited expression, it also prevents verification of demographic information such as location, identity, or political orientation. The discourse may skew towards ideological or socioeconomic groups—such as liberal-leaning or college-educated users—that are disproportionately active on the platform. Claims about representativeness should therefore be made cautiously.

Second, purposive sampling of high-engagement threads, while methodologically justified, may overrepresent extreme or performative sentiments. Viral comments risk being misread as majority opinion rather than as highly visible outliers. Future studies could complement interpretive approaches with computational methods—such as sentiment analysis or network mapping—to assess the broader distribution of discursive themes across the platform.

Third, this study does not address offline behaviour or real-world action. The folk justice documented here remains largely symbolic. Ethnographic or interview-based research could usefully explore whether online sentiments translate into civic or political engagement beyond the digital sphere.

Fourth, Reddit’s content moderation policies, subreddit cultures, and user demographics are subject to rapid change. Longitudinal studies would help track how digital moral sentiment emerges, evolves, or dissipates—particularly around high-profile trials or state interventions.

Finally, while the counter-public framework applied here draws on the Fraserean tradition, deeper engagement with the broader counter-public literature remains a direction for future theoretical development.

## Data Availability

The original contributions presented in the study are included in the article/supplementary material, further inquiries can be directed to the corresponding author.
